# A novel nomogram for identifying candidates for adjuvant chemotherapy in patients with stage IB gastric adenocarcinoma

**DOI:** 10.1186/s12876-023-02706-6

**Published:** 2023-03-06

**Authors:** Yangyang Xie, Xue Song, Danwei Du, Haimin Jin, Xiaowen Li, Zhongkai Ni, Hai Huang

**Affiliations:** 1 Department of General Surgery, Hangzhou TCM Hospital Affiliated to Zhejiang Chinese Medical University, #453, Tiyuchang Road, Xihu District, Hangzhou, 310000 Zhejiang Province China; 2 Department of Respiratory and Critical Care Medicine, Hangzhou TCM Hospital Affiliated to Zhejiang Chinese Medical University, #453, Tiyuchang Road, Xihu District, Hangzhou, 310000 Zhejiang Province China

**Keywords:** Stage IB gastric cancer, SEER Program, Chemotherapy, Nomogram

## Abstract

**Background:**

The purpose of this research was to construct a novel predictive nomogram to identify specific stage IB gastric adenocarcinoma (GAC) populations who could benefit from postoperative adjuvant chemotherapy (ACT).

**Method:**

Between 2004 and 2015, 1889 stage IB GAC patients were extracted from the Surveillance, Epidemiology, and End Results (SEER) program database. Then Kaplan–Meier survival analysis, univariate and multivariable Cox analyses, and univariate and multivariable logistic analyses were implemented. Finally, the predictive nomograms were constructed. The methods of area under the curve (AUC), calibration curve, and decision curve analysis (DCA) were used to validate the clinical effectiveness of the models.

**Results:**

Of these patients, 708 cases underwent ACT, while the other 1181 patients didn’t receive ACT. After PSM, the patients in the ACT group presented a longer median overall survival (133 vs. 85 months, *p* = 0.0087). Among the ACT group, 194 (36.0%) patients achieving more prolonged overall survival than 85 months were regarded as the beneficiary population. Then the logistic regression analyses were performed, and age, gender, marital status, primary site, tumor size, and regional nodes examined were included as predicting factors to construct the nomogram. The AUC value was 0.725 in the training cohort and 0.739 in the validation cohort, which demonstrated good discrimination. And calibration curves indicated ideal consistency between the predicted and observed probabilities. Decision curve analysis presented a clinically useful model. Furthermore, the prognostic nomogram predicting 1-, 3-, and 5-year cancer-specific survival presented good predictive ability.

**Conclusion:**

The benefit nomogram could guide clinicians in decision-making and selecting optimal candidates for ACT among stage IB GAC patients. And the prognostic nomogram presented great prediction ability for these patients.

**Supplementary Information:**

The online version contains supplementary material available at 10.1186/s12876-023-02706-6.

## Introduction

Gastric cancer (GC) is one of the most common malignancies and the third leading cause of mortality from tumors globally [1]. And gastric adenocarcinoma (GAC) is the most common subtype of GC [2]. Currently, the strategies of postoperative adjuvant therapy are mainly derived from the ACTS-GC trial [3] and the CLASSIC trial [4]. In stage II-III GC patients following radical surgery, adjuvant chemotherapy (ACT) has been shown to be beneficial. However, the role of ACT in stage IB GC patients is not well specified yet. According to the National Comprehensive Cancer Network (NCCN) guidelines, high-risk T2N0M0 and T1N1M0 (invading blood vessel, younger than 50 years, poorly differentiated subtype, et al.) patients are most likely to benefit from ACT after the radical operation [5]. ACT was also effective for stage T2N0M0 GC and stage T1a/1bN1M0 GC after D2 gastrectomy, according to the European guideline [6]. Based on the Japanese Gastric Cancer Treatment Guidelines, on the other hand, a close follow-up alone is recommended for stage I patients [7].

Despite the controversial role of ACT in stage IB GC therapy, it’s clear that a specific group of these patients can obtain more prolonged survival after ACT. Identifying candidates who could gain potential benefits from ACT is an urgent issue that needs to be addressed.

The retrospective research aimed to use the Surveillance, Epidemiology, and End Results (SEER) database to construct an effective model to identify specific stage IB GAC populations who could benefit from ACT. And a prognostic nomogram was developed to predict the survival of these patients.

## Materials and methods

### Data source and patient selection

Population-based information was retrieved from the SEER program. The inclusion criteria were as follows: (1) age older than 18 years; (2) pathologic confirmation was adenocarcinoma, mucinous adenocarcinoma, mucin-producing adenocarcinoma, mucinous cyst-adenocarcinoma, signet ring cell carcinoma, papillary adenocarcinoma, tubular adenocarcinoma, adenocarcinoma intestinal type, carcinoma diffuse type, adenocarcinoma with mixed subtype; (3) patients who received radical operation; (4) the sixth edition AJCC stage was IB (T1N1M0 or T2aN0M0).

**Fig. 1:**
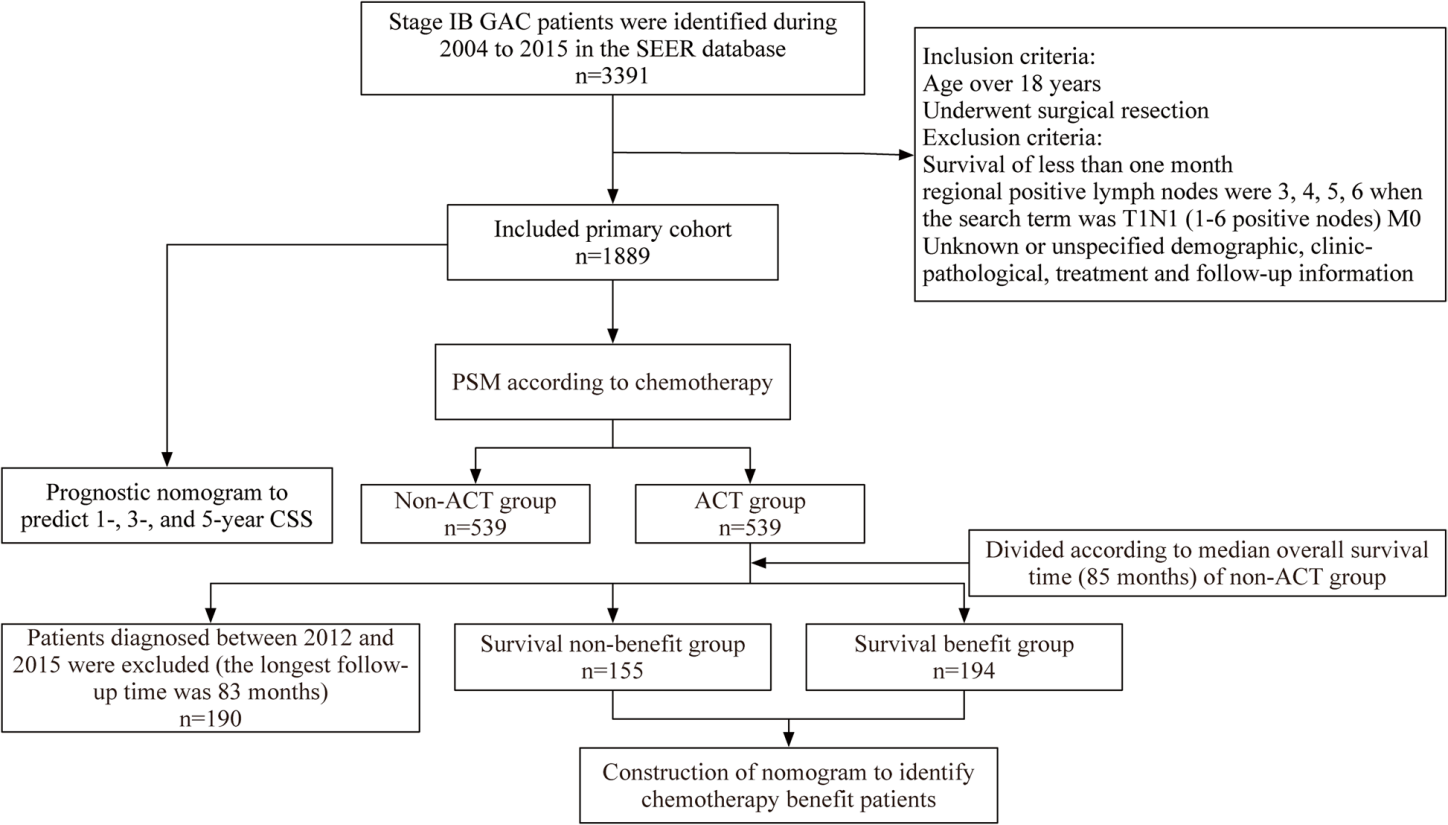
The workflow of the patient selection process

The exclusion criteria were as follows: (1) patients who only lived for a month or less; (2) regional positive lymph nodes were 3, 4, 5, and 6 among T1N1 (1–6 positive nodes) M0 patients because all these data were translated to conform the eighth edition of the AJCC system to get a sufficient follow-up time; (3) Patients with incomplete demographic, clinicopathological, therapy or follow-up data were eliminated from the study. In the end, 1889 patients were enrolled in the research. The process of patient selection is presented in Fig. 1.

### Data collection

Demographic data included year at diagnosis, age at diagnosis, gender, race, marital status, grade, pathology, primary site, tumor size, regional nodes examined (RNE), stage status, chemotherapy, and prognostic information. Patients were categorized according to the primary site (cardia, distal site, middle site and overlapping/NOS), tumor size (≤ 2 cm, ≤ 5 cm, and > 5 cm), RNE (1–15 and ≥ 16), and stage (T1N1M0 and T2N0M0). The variable of “RX Summ–Systemic/Sur Seq” in raw data was used to distinguish neoadjuvant and adjuvant chemotherapy. The stage IB GAC patients were reclassified using the eighth edition of the AJCC staging system. Based on the median OS of the non-ACT cohort (85 months), ACT patients were divided into the benefit group (surviving more than 85 months) and the non-benefit group (surviving less than 85 months). We defined that these patients in the benefit group could benefit from ACT.

### Statistical analysis

The survival analysis was performed by the Kaplan–Meier approach and log-rank test was used to explore the survival difference. Then we performed subgroup analyses using the univariate Cox proportional hazard model to calculate the hazard ratios (HRs) of the two cohorts in specific patient subgroups. Forest plots were developed to show every parameter’s effect on OS. HRs and 95% confidential intervals (CIs) were recorded.

The propensity score matching (PSM) method was a useful statistical technique for reducing confounding and simulating randomized controlled trials [8]. By using logistic regression, all factors were used to obtain a propensity score. The one-to-one nearest-neighbor technique on the logit scale was used to match cases in the two cohorts (ACT and non-ACT). The caliper was calibrated to 0.01. The change in variables before and after PSM was presented using standardized difference (SD). SD less than 0.1 meant that the baseline parameters were in ideal balance [9]. The cases in the ACT group were then randomly separated into a training group (70%, *n* = 244) and a validation group (30%, *n* = 105) for further investigation.

To find independent determinants for ACT benefit likelihood, logistic regression analysis was performed. The variables with a *p*-value smaller than 0.2 were used for the multivariable analysis after the univariate analysis. The factors screened out by multiple logistic regression models (*P* < 0.05) were included. Then a predictive nomogram was developed to identify potential ACT-beneficial cases. In the model, a vertical line is drawn to each parameter’s “points" line, and the sum of every point corresponds to the benefit probability. Using the “benefit” logistic regression model, we could predict the probability of the occurrence of “benefit”. That was, the probability that patients could benefit from ACT. The stage IB GAC cases with a benefit probability of over 50% were regarded as candidates for ACT benefit. Besides, the prognostic elements identified in the multivariable Cox regression were included to develop 1-, 3-, and 5-year cancer-specific survival (CSS) nomograms in the training dataset.

The area under the receiver operating characteristics curve (AUC) and calibration curves were used to assess the performance of the nomogram in the training and validation groups, respectively. 1000 bootstrap resamples were run on the calibration curves to see if the predicted and observed survival probabilities were consistent. The receiver operating characteristic (ROC) curves were also used to calculate AUC and emphasize the constructed model's prediction power. The prediction power of an AUC with a higher value was greater. Finally, decision curve analysis (DCA) was employed to calculate the net benefit for a group of threshold probabilities, allowing researchers to assess the nomogram's practicability for guiding clinical decisions [10]. The X-Tile software was used to develop a novel risk stratification system based on the best risk score cutoff value, classifying patients into low-, middle-, and high-risk groups.

The analyses above were consistent with NCI statements about the reliability of the chemotherapy data. R software (version 4.1.2, The R Foundation for Statistical Computing, Vienna, Austria; http://www.r-project.org) was used for all statistical analyses and visualizations. It was determined that a two-tailed *P* < 0.05 was statistically significant.

## Results

### Patients' demographics

From 2004 to 2015, 1889 stage IB GAC patients were recruited. 708 patients had undergone ACT treatment, while the remaining 1181 had not. Year of diagnosis, age, gender, marital status, grade, pathology, primary site, tumor size, RNE, and stage status were all shown to be significantly different between the two groups (*P* < 0.05). The patients who received ACT presented higher proportion of 2012–2015 period (39.0% vs. 25.7%), male (67.8% vs. 60.9%), married status (68.2% vs. 60.8%), III/IV grade (60.7% vs. 51.9%), SRCC pathology (17.4% vs. 13.0%), cardia tumor (39.3% vs. 22.2%), RNE ≥ 16 (43.1% vs. 36.7%), T1N1M0 stage (45.5% vs. 21.8%). The non-ACT group presented more percentage when the tumor size > 5 cm (16.8% vs. 13.8%).

PSM was used to minimize selection bias and balanced the distribution of potential confounders due to unmatched parameters across the two cohorts. Most variables had SDs < 0.1 after PSM, indicating ideal balance (Figure S1). Finally, 1078 patients were separated into two groups: those with ACT (*n* = 539) and those without ACT (*n* = 539). Demographic for patients in two sets before and after PSM are shown in Table 1.

**Table 1:** The characteristics of stage IB GAC patients before and after PSM

Characteristics	Before PSM				After PSM			
	All	ACT	Non-ACT	*P* value	All	ACT	Non-ACT	*P* value
	*N* = 1889	*N* = 708	*N* = 1181		*N* = 1078	*N* = 539	*N* = 539	
Year at diagnosis:				< 0.001				0.446
2004–2007	700 (37.1%)	215 (30.4%)	485 (41.1%)		362 (33.6%)	173 (32.1%)	189 (35.1%)	
2008–2011	609 (32.2%)	217 (30.6%)	392 (33.2%)		354 (32.8%)	176 (32.7%)	178 (33.0%)	
2012–2015	580 (30.7%)	276 (39.0%)	304 (25.7%)		362 (33.6%)	190 (35.3%)	172 (31.9%)	
Age	68.4 (12.3)	62.4 (11.3)	72.0 (11.5)	< 0.001	64.9 (10.7)	64.9 (10.3)	65.0 (11.1)	0.822
Gender:				0.003				0.365
Female	690 (36.5%)	228 (32.2%)	462 (39.1%)		357 (33.1%)	186 (34.5%)	171 (31.7%)	
Male	1199 (63.5%)	480 (67.8%)	719 (60.9%)		721 (66.9%)	353 (65.5%)	368 (68.3%)	
Race:				0.091				0.897
White	1245 (65.9%)	484 (68.4%)	761 (64.4%)		725 (67.3%)	361 (67.0%)	364 (67.5%)	
Non-White	644 (34.1%)	224 (31.6%)	420 (35.6%)		353 (32.7%)	178 (33.0%)	175 (32.5%)	
Marital status:				0.001				0.795
Married	1201 (63.6%)	483 (68.2%)	718 (60.8%)		727 (67.4%)	366 (67.9%)	361 (67.0%)	
Unmarried	688 (36.4%)	225 (31.8%)	463 (39.2%)		351 (32.6%)	173 (32.1%)	178 (33.0%)	
Grade:				< 0.001				0.621
I/II	846 (44.8%)	278 (39.3%)	568 (48.1%)		449 (41.7%)	220 (40.8%)	229 (42.5%)	
III/IV	1043 (55.2%)	430 (60.7%)	613 (51.9%)		629 (58.3%)	319 (59.2%)	310 (57.5%)	
Pathology:				0.01				0.736
Non-SRCC	1613 (85.4%)	585 (82.6%)	1028 (87.0%)		911 (84.5%)	458 (85.0%)	453 (84.0%)	
SRCC	276 (14.6%)	123 (17.4%)	153 (13.0%)		167 (15.5%)	81 (15.0%)	86 (16.0%)	
Primary site:				< 0.001				0.888
Cardia	540 (28.6%)	278 (39.3%)	262 (22.2%)		367 (34.0%)	183 (34.0%)	184 (34.1%)	
Distal site	603 (31.9%)	179 (25.3%)	424 (35.9%)		294 (27.3%)	152 (28.2%)	142 (26.3%)	
Middle site	547 (29.0%)	191 (27.0%)	356 (30.1%)		314 (29.1%)	155 (28.8%)	159 (29.5%)	
Overlapping/NOS	199 (10.5%)	60 (8.5%)	139 (11.8%)		103 (9.6%)	49 (9.1%)	54 (10.0%)	
Tumor size:				< 0.001				0.782
≤ 2 cm	542 (28.7%)	216 (30.5%)	326 (27.6%)		319 (29.6%)	162 (30.1%)	157 (29.1%)	
≤ 5 cm	908 (48.1%)	308 (43.5%)	600 (50.8%)		506 (46.9%)	247 (45.8%)	259 (48.1%)	
> 5 cm	296 (15.7%)	98 (13.8%)	198 (16.8%)		163 (15.1%)	81 (15.0%)	82 (15.2%)	
Unknown	143 (7.6%)	86 (12.1%)	57 (4.8%)		90 (8.3%)	49 (9.1%)	41 (7.6%)	
RNE:				0.007				0.39
≥ 16	738 (39.1%)	305 (43.1%)	433 (36.7%)		471 (43.7%)	228 (42.3%)	243 (45.1%)	
1–15	1151 (60.9%)	403 (56.9%)	748 (63.3%)		607 (56.3%)	311 (57.7%)	296 (54.9%)	
Stage:				< 0.001				0.849
T1N1M0	579 (30.7%)	322 (45.5%)	257 (21.8%)		384 (35.6%)	194 (36.0%)	190 (35.3%)	
T2N0M0	1310 (69.3%)	386 (54.5%)	924 (78.2%)		694 (64.4%)	345 (64.0%)	349 (64.7%)	

### Survival analysis

**Fig. 2:**
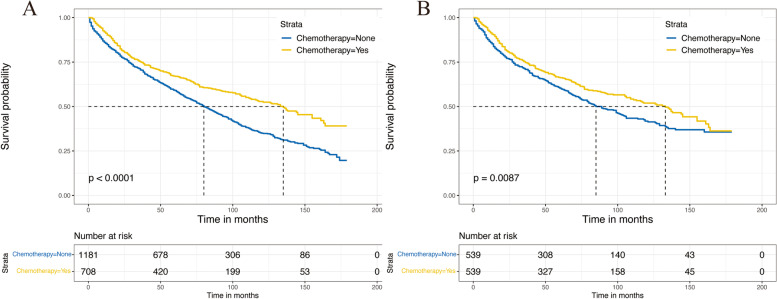
Overall survival curves in the ACT group and non-ACT group. **A** Before PSM. **B** After PSM

**Fig. 3:**
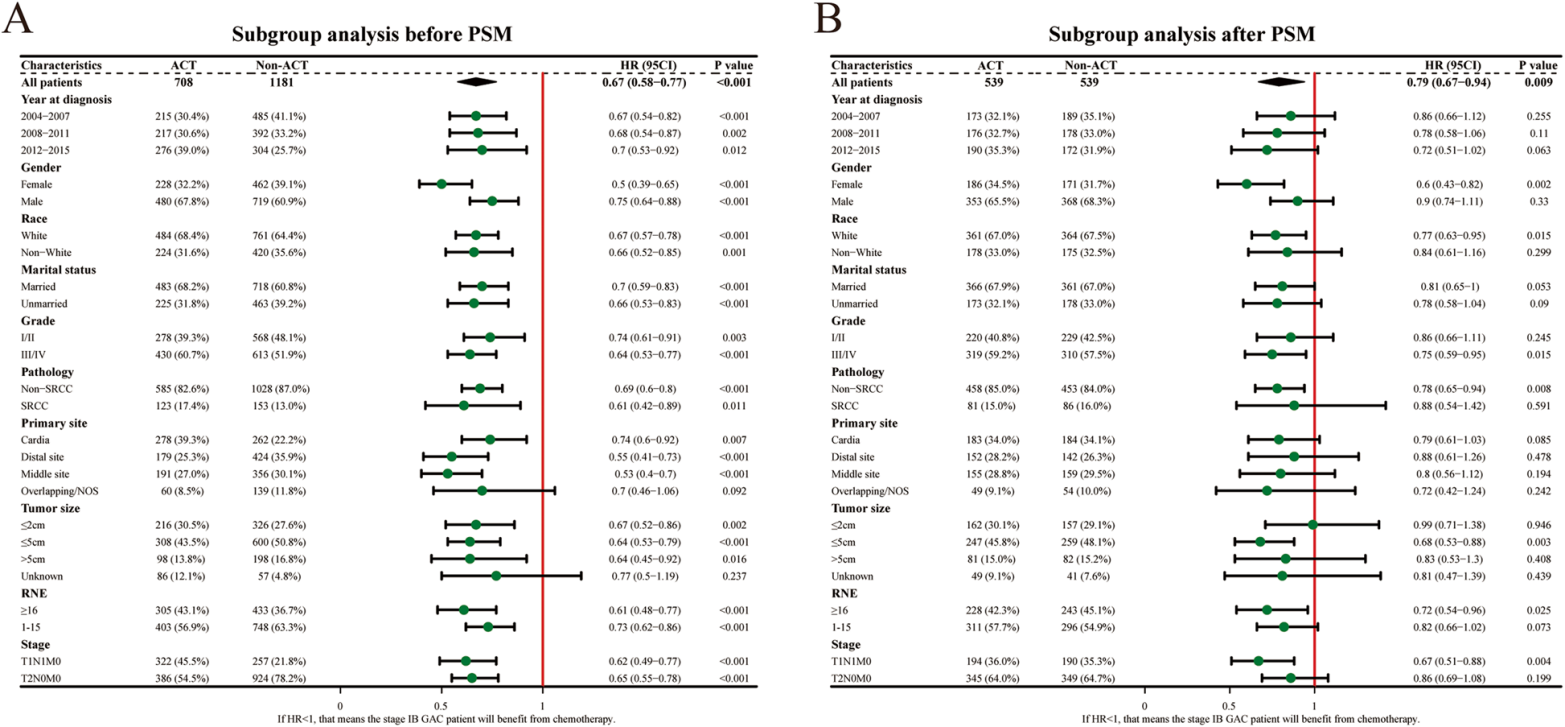
Forest plot of hazard ratios (HRs) for overall survival in the two groups. Diamonds represent effect size, calculated separately in different subgroups, and error bars indicate 95% CIs. **A** Before PSM. **B** After PSM

In the Kaplan–Meier analysis, a significant difference in OS was found in two groups at the pre- and post-match stages. Before PSM, the patients who underwent ACT presented longer median OS than the non-ACT cohort (135 vs. 80 months, *p* < 0.001) (Fig. 2A). After PSM, a similar result was observed (133 vs. 85 months, *p* = 0.0087) (Fig. 2B). In the forest plots, HRs in all subgroups were less than one before and after PSM, indicating that these patients could benefit from ACT in all subgroups (Fig. 3). Then 190 ACT patients diagnosed between 2013 and 2015 (the longest follow-up time as 83 months) were excluded for further study because we couldn’t specify if these patients occur the event of interest.

### Constructing and verifying the benefit nomogram

The cases in the ACT group were then randomly separated into a training group (70%, *n* = 244) and a validation group (30%, *n* = 105) for further investigation. Table S1 shows the essential features of the two groups. To identify independent components, univariate and multivariable logistic regression analyses were used (Table 2). Age, gender, marital status, primary site, tumor size, and RNE were found to be independent predictors of the beneficial probability of stage IB GAC patients receiving ACT.

**Table 2:** Logistic regression analysis of the significant factors for ACT benefit

Characteristics	Univariate analysis			Multivariate analysis		
	OR	95%CI	*P* value	OR	95%CI	*P* value
Age	0.96	0.94–0.99	< 0.001	0.97	0.94–0.99	0.016
Gender:						
Female	Reference			Reference		
Male	0.58	0.34–0.99	0.04	0.54	0.26–0.95	0.039
Race:						
White	Reference			Reference		
Non-White	1.48	0.87–2.53	0.15	0.96	0.51–1.82	0.911
Marital status:						
Married	Reference			Reference		
Unmarried	0.59	0.34–1.04	0.07	0.42	0.22–0.81	0.009
Grade:						
I/II	Reference			Reference		
III/IV	1.43	0.85–2.4	0.17	1.01	0.55–1.86	0.965
Pathology:						
Non-SRCC	Reference			Reference		
SRCC	1.25	0.64–2.47	0.51			
Primary site:						
Cardia	Reference			Reference		
Distal site	2.75	1.43–5.32	< 0.001	2.33	1.07–5.1	0.034
Middle site	2.28	1.17–4.45	0.02	1.75	0.8–3.84	0.163
Overlapping/NOS	1.88	0.69–5.11	0.22	1.4	0.46–4.31	0.557
Tumor size:						
≤ 2 cm	Reference			Reference		
≤ 5 cm	0.95	0.53–1.69	0.85	0.99	0.52–1.87	0.97
> 5 cm	1.01	0.45–2.29	0.98	0.88	0.36–2.15	0.772
Unknown	0.4	0.14–1.11	0.08	0.39	0.13–0.93	0.007
RNE:						
≥ 16	Reference			Reference		
1–15	0.52	0.31–0.88	0.02	0.47	0.27–0.84	0.01
Stage:						
T1N1M0	Reference			Reference		
T2N0M0	0.96	0.58–1.61	0.89			

**Fig. 4:**
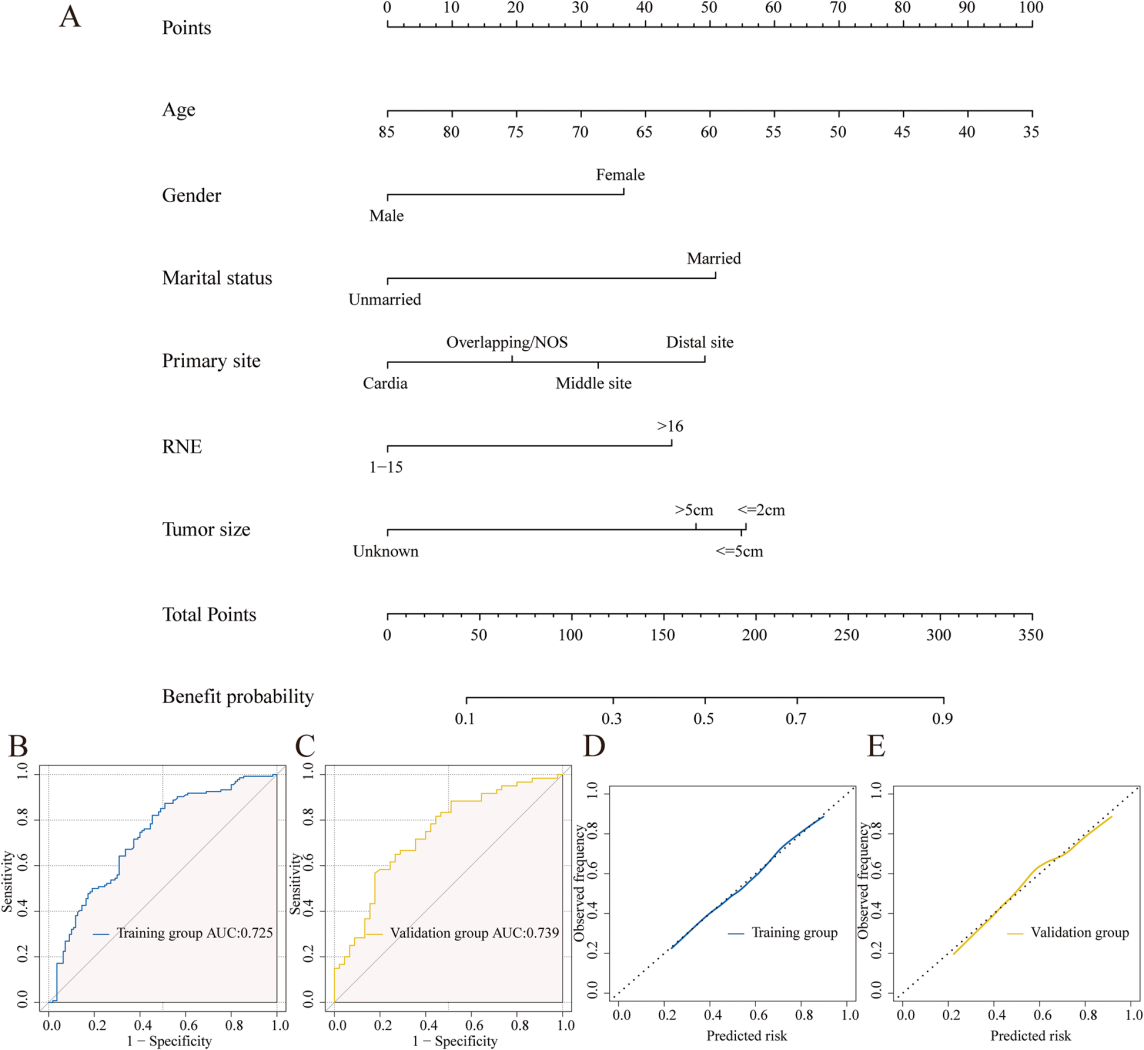
The benefit nomogram to identify optimal candidates among stage IB GAC patients who could obtain survival benefits from chemotherapy (**A**). The calculated points correspond to a benefit probability. ROC curves of the nomogram in the training (**B**) and validation (**C**) cohort. Calibration curves in the training (**D**) and validation (**E**) cohort

A predictive nomogram was constructed to identify potential ACT-beneficial cases based on the multivariable logistic regression model (Fig. 4A). The total score was calculated by summing the scores corresponding to the six parameters. Then the model could be used to predict the beneficial probability of ACT.

The AUCs of the nomogram were 0.725 and 0.739 in training cohort and validation cohort, which presented good discriminatory power in both groups (Fig. 4B and 4C). We also utilized calibration plots to verify the model's prediction accuracy; the findings showed that the anticipated and observed probabilities were perfectly consistent (Fig. 4D and 4E). The nomogram's good clinical practical utility was next confirmed by DCA curves in both sets (Figures S2A and S2B). The results demonstrated our nomogram's excellent predictive potential as well as its high trustworthiness.

### Constructing and verifying the prognostic nomogram

**Fig. 5:**
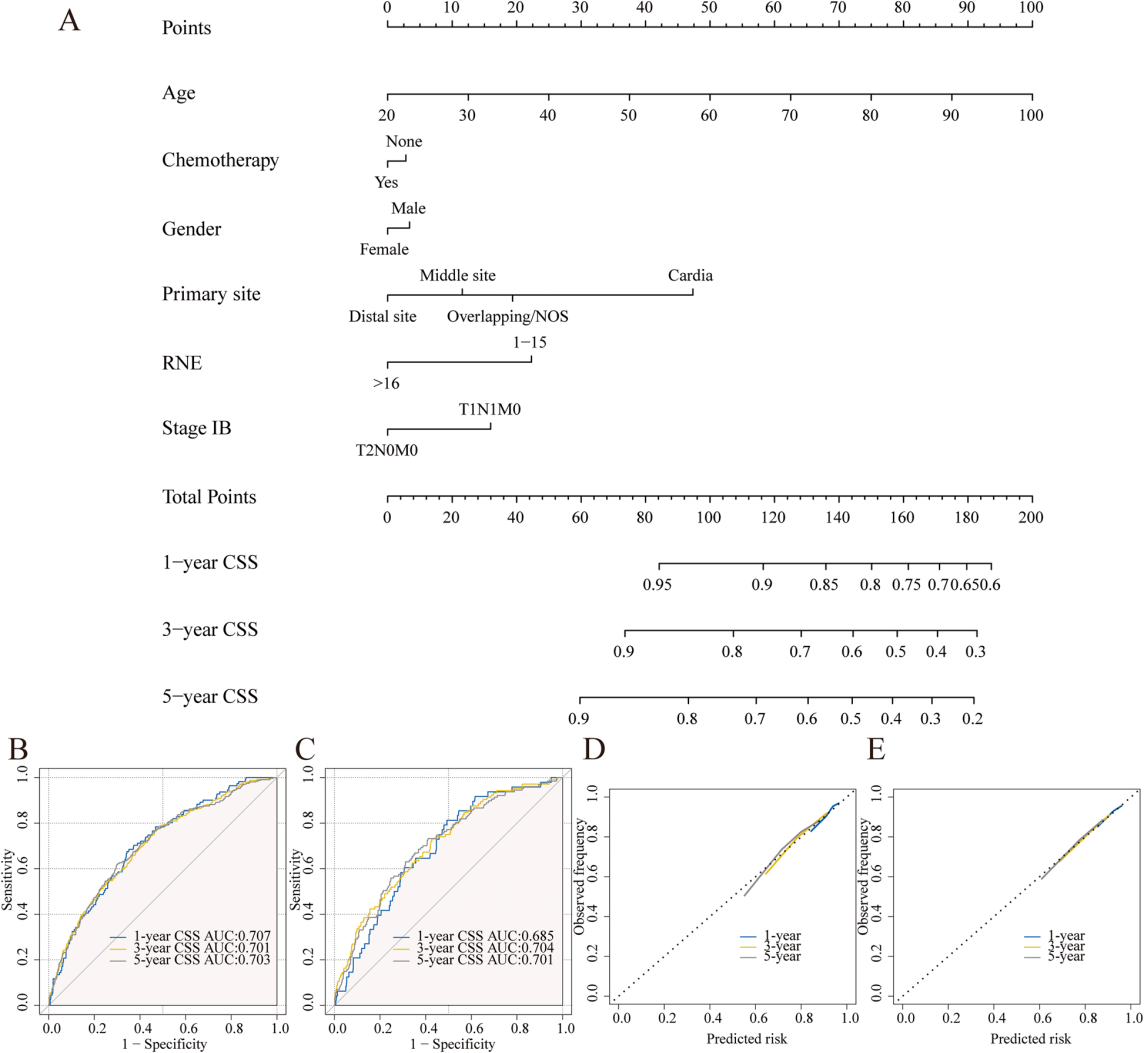
The prognostic nomogram to predict 1-, 3-, and 5-year CSS among stage IB GAC patients (**A**). ROC curves of the nomogram in the training (**B**) and validation (**C**) cohort. Calibration curves in the training (**D**) and validation (**E**) cohort

Subsequently, a second and independent investigation was performed. We randomly separated the overall cases into a training set (70%, *n* = 1322) and a validation set (30%, *n* = 567) with the goal of creating a nomogram to predict 1-, 3-, and 5-year CSS in stage IB GAC patients. Then, using multivariable Cox regression, the significant variables (*P* < 0.2) revealed by univariate Cox regression were further discovered, revealing that age, gender, stage, RNE, and ACT were all independent variables (Table 3, Fig. 5A).

**Table 3:** Univariate and Multivariate Cox regression analysis of CSS in stage IB GAC patients in the training cohort

Characteristics	Univariate analysis			Multivariate analysis		
	HR	95%CI	P	HR	95%CI	P
Age	1.03	1.02–1.04	< 0.001	1.03	1.02–1.04	< 0.001
Gender						
Female	Reference			Reference		
Male	1.34	1.08–1.67	0.008	1.25	1.05–1.57	0.048
Race						
White	Reference			Reference		
Non-White	0.74	0.59–0.92	0.007	1.05	0.83–1.33	0.683
Marital status						
Married	Reference					
Unmarried	1.05	0.85–1.29	0.675			
Grade						
I/II	Reference			Reference		
III/IV	0.81	0.66–0.99	0.038	0.97	0.78–1.2	0.777
Pathology						
Non-SRCC	Reference			Reference		
SRCC	0.81	0.6–1.1	0.175	1.23	0.88–1.72	0.218
Primary site						
Cardia	Reference			Reference		
Distal site	0.4	0.31–0.52	< 0.001	0.33	0.24–0.44	< 0.001
Middle site	0.51	0.39–0.66	< 0.001	0.45	0.34–0.59	< 0.001
Overlapping/NOS	0.74	0.53–1.04	0.088	0.62	0.43–0.88	0.008
Tumor size						
≤ 2 cm	Reference			Reference		
≤ 5 cm	1.22	0.96–1.56	0.107	1.16	0.91–1.49	0.226
> 5 cm	1.01	0.71–1.42	0.97	1.25	0.88–1.77	0.215
Unknown	1.46	1–2.12	0.048	1.38	0.94–2.01	0.098
RNE						
≥ 16	Reference			Reference		
1–15	1.55	1.24–1.93	< 0.001	1.53	1.22–1.92	< 0.001
Stage						
T1N1M0	Reference			Reference		
T2N0M0	0.74	0.6–0.91	0.004	0.7	0.56–0.87	0.001
Chemotherapy						
No	Reference			Reference		
Yes	0.76	0.61–0.94	0.011	0.77	0.6–0.98	0.032

The validity of the model was then confirmed using the validation cohort. The training cohort's 1-, 3-, and 5-year AUC values were 0.707, 0.701, and 0.703, respectively, whereas the validation cohort's AUC values were 0.685, 0.704, and 0.701. (Fig. 5B and 5C). The high AUC values suggested that the discrimination capacity was good. We also utilized calibration plots to test the model's prediction accuracy and found that the predicted and observed survival probabilities were rather consistent (Figs. 5D and 5E). DCA curves in both groups proved the nomogram's strong clinical practical value (Figure S2C-H). The results demonstrated our nomogram's excellent predictive potential as well as its high trustworthiness.

**Fig. 6:**
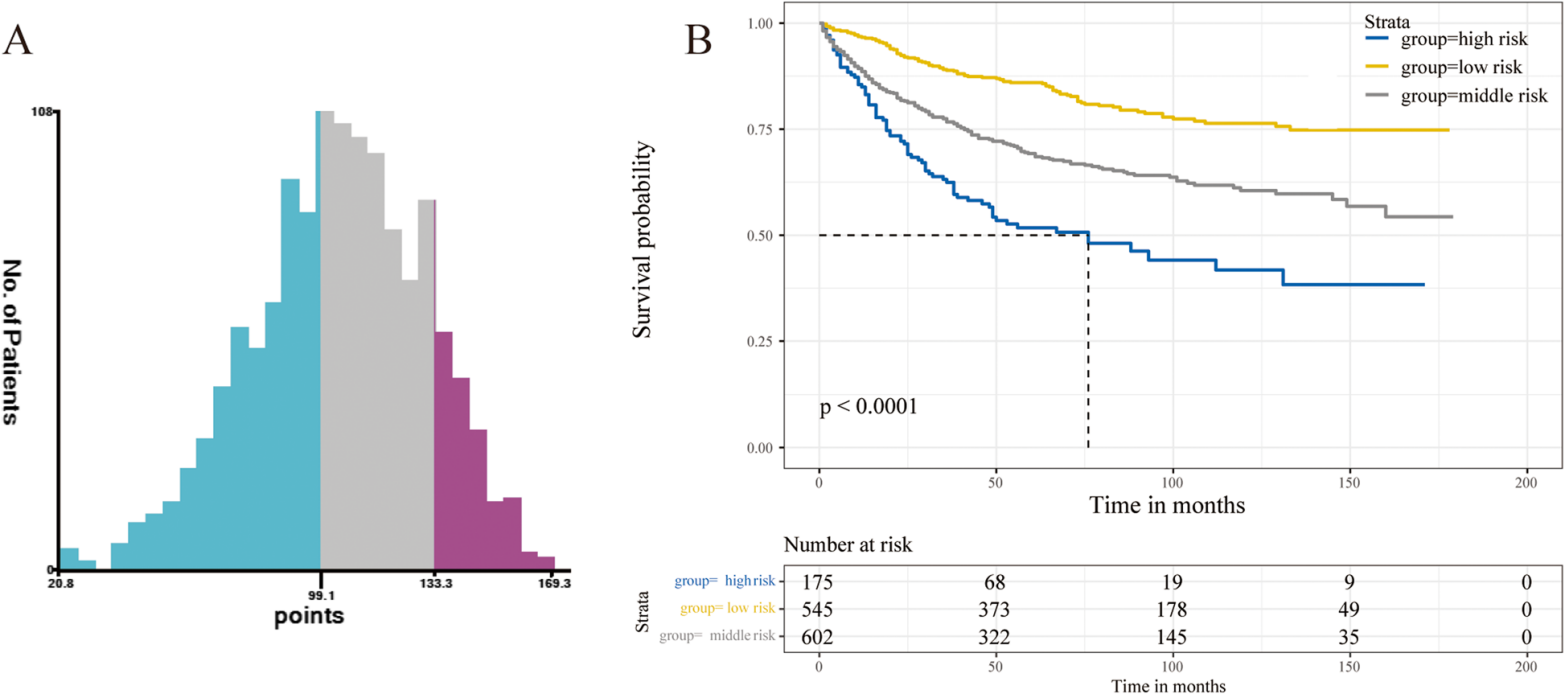
**A** Cut-off point for risk stratification using X-tile. **B** KM curves based on the three risk categories

Based on the analysis of X-tile software, patients were separated into three risk cohorts, including low risk ( total points < 99), middle risk (99 ≤ total points < 113), and high risk (total points ≥ 113; Fig. 6A). Significant discrimination in the three risk categories was shown by KM curves (Fig. 6B).

## Discussion

This study indicated that the stage IB GAC patient who underwent ACT presented longer survival than the non-ACT cases. A predictive nomogram was constructed to identify the specific populations that would benefit from chemotherapy. The nomogram incorporating age, gender, marital status, primary site, tumor size, and RNE presented potential clinical applicability. To the authors' knowledge, this is the first study to develop a unique nomogram for identifying potential populations who may benefit from ACT.

Several randomized clinical trials (RCTs) have focused on the role of chemotherapy among GC patients. Stage II/III GC patients who had adjuvant chemoradiotherapy presented a better overall survival and relapse-free survival in the SWOG-directed INT-0116 study [11]. MAGIC [12], ACTS-GC [13], and CLASSIC [4] trials also presented that majority of high-stage cases could benefit from ACT. Nonetheless, it remained unclear whether all stages of GC (particularly stage IB GC) that received ACT would improve the prognosis. Furthermore, the administration of ACT to stage IB GC has been a source of contention in many regional guidelines [5, 7, 14]. The European and NCCN guidelines recommended ACT for stage IB GC patients after radical surgery [6]. The Japanese guideline, on the other hand, only advised a close follow-up approach for stage I patients [7].

In the absence of relevant RCTs, a series of retrospective investigations have focused on stage IB GC. Seyedin et al. indicated that adjuvant treatment could prolong survival compared with the surgery-only patient [15]. Furthermore, according to a recent study based on the National Cancer Database, stage IB patients who receive adjuvant chemoradiotherapy have a considerably decreased risk-adjusted mortality rate [16]. However, a Korean research found no advantage from ACT in terms of disease-free survival or tumor recurrence among stage IB GC cases, supporting the recommendations of the Japanese guideline [17]. Dudeja et al. enrolled nearly 2000 postoperative GC patients, finding that the subgroup with early-stage (stage I-T1/T2, N0, or T1/N1) might benefit from ACT [18]. Despite these conflicting results, it was clear that a specific population of stage IB GAC patients could benefit from ACT. Wang et al. divided the stage IB GC patients into low-risk and high-risk cohorts based on the projected 5-year OS of recursive partitional analyses, indicating that ACT was only recommended for high-risk patients [19]. However, this method could only distinguish a group of high-risk patients without definite probability. The nomogram proposed in our study could calculate each patient’s risk points and predict the personal beneficial probability of ACT.

In our visualized nomogram, age, gender, marital status, primary site, tumor size, and RNE were primary predictive variables, providing individualized estimates of whether stage IB GAC patients could benefit from the ACT. The older patients would probably suffer a low beneficial probability than young patients. And female and married patients have a higher beneficial probability than male and unmarried populations. In addition, the model also showed the beneficial effect of the RNE ≥ 16 cases. One reason could be that removing more lymph nodes would increase the likelihood of detecting metastatic lymph nodes and contribute to improving nodal staging accuracy. Some patients with the N0 stage might have a node-positive condition. This group of patients was less likely to receive ACT due to the underestimated tumor stage.

The nomogram was assessed in the training and validation set. The AUCs (0.725 and 0.739) and calibration curves presented reliable discrimination and calibration ability. Moreover, the DCA analysis confirmed the excellent applicability of the model. Certain individual conditions are crucial to selecting optimal candidates for ACT among stage-IB patients. The combination of multiple predicting factors could provide a more reliable prediction than any simple single indicator. The predictive model allows clinicians to calculate each patient’s total points and beneficial probability. Thus, this exploratory study built an individualized prediction nomogram to identify ACT benefit candidates, which could assist clinicians in decision-making.

Meanwhile, age, gender, stage, RNE, and chemotherapy were used to build a relatively reliable and discriminating prognostic nomogram for predicting 1-, 3-, and 5-year CSS in stage IB GAC patients. In addition, the model had a high level of predictability and credibility. Then, applying X-tile software to determine the cutoff value for the best grouping, we created a novel risk stratification system that separated all cases into low-, middle-, and high-risk groups and demonstrated a remarkable ability to distinguish different risk groups.

### Limitation

The study yielded solid results in terms of identifying stage IB GAC patients who would benefit from treatment. However, there were some flaws in the current study that should be addressed. To begin with, the lack of specific chemotherapy information made it difficult to compare the effects of various chemotherapy medicines. Second, the application of PSM indicated that the nomogram was only relevant to people who were similar to those included in the propensity score analysis. Finally, because this was a retrospective analysis, so selection bias was inevitably introduced.

## Conclusion

Our research indicated that stage IB GAC patients who underwent ACT presented longer median OS than the non-ACT cohort. The high-performing nomogram could guide surgeons in decision-making and selecting optimal candidates for the ACT. And the prognostic nomogram predicted the individualized probability of CSS at 1-, 3-, and 5-year presenting good prediction ability. Further research and RCTs are required to validate the conclusion.

## Supplementary Information

**Additional file 1:** **Table S1.** The basic characteristics of stage IB GAC patients in the training and validation group. **Figure S1.** The mean difference between the two cohorts. **Figure S2.** DCA curves of the benefit nomogram in the training (A) and validation (B) cohort. DCA curves of the prognostic nomogram in the training (C-E) and validation (F-H).

## Data Availability

The datasets created and analyzed during this investigation are available in the SEER database (https://seer.cancer.gov/). Hai Huang could be contacted upon reasonable requestion for the data from the study.
