# Supplementary material for: A novel nomogram for identifying candidates for adjuvant chemotherapy in patients with stage IB gastric adenocarcinoma
Source: BMC Gastroenterol. 2023 Mar 6;23:54. doi: 10.1186/s12876-023-02706-6 (PMC9987131; doi:10.1186/s12876-023-02706-6)
Supplement: Supplementary file 1 — Additional file 1: Table S1. The basic characteristics of stage IB GAC patients in the training and validation group. Figure S1. The mean difference between the two cohorts. Figure S2. DCA curves of the benefit nomogram in the training (A) and validation (B) cohort. DCA curves of the prognostic nomogram in the training (C-E) and validation (F-H). [file 12876_2023_2706_MOESM1_ESM.docx]

| Table S1. The basic characteristics of stage IB GAC patients in the training and validation group | | | | |
| --- | --- | --- | --- | --- |
| Characteristics | All | Training | Validation | P value |
|  | N=349 | N=105 | N=244 |  |
| Year at diagnosis: |  |  |  | 0.082 |
| 2004-2007 | 173 (49.6%) | 60 (57.1%) | 113 (46.3%) |  |
| 2008-2011 | 176 (50.4%) | 45 (42.9%) | 131 (53.7%) |  |
| Age | 64.3 (10.7) | 65.3 (11.0) | 63.9 (10.6) | 0.291 |
| Gender: |  |  |  | 0.392 |
| Female | 123 (35.2%) | 33 (31.4%) | 90 (36.9%) |  |
| Male | 226 (64.8%) | 72 (68.6%) | 154 (63.1%) |  |
| Race: |  |  |  | 0.4 |
| White | 233 (66.8%) | 74 (70.5%) | 159 (65.2%) |  |
| Non-White | 116 (33.2%) | 31 (29.5%) | 85 (34.8%) |  |
| Marital status: |  |  |  | 0.682 |
| Married | 249 (71.3%) | 77 (73.3%) | 172 (70.5%) |  |
| Unmarried | 100 (28.7%) | 28 (26.7%) | 72 (29.5%) |  |
| Grade: |  |  |  | 0.978 |
| I/II | 135 (38.7%) | 40 (38.1%) | 95 (38.9%) |  |
| III/IV | 214 (61.3%) | 65 (61.9%) | 149 (61.1%) |  |
| Pathology: |  |  |  | 0.092 |
| Non-SRCC | 297 (85.1%) | 95 (90.5%) | 202 (82.8%) |  |
| SRCC | 52 (14.9%) | 10 (9.5%) | 42 (17.2%) |  |
| Primary site: |  |  |  | 0.072 |
| Cardia | 109 (31.2%) | 38 (36.2%) | 71 (29.1%) |  |
| Distal site | 102 (29.2%) | 21 (20.0%) | 81 (33.2%) |  |
| Middle site | 105 (30.1%) | 33 (31.4%) | 72 (29.5%) |  |
| Overlapping/NOS | 33 (9.5%) | 13 (12.4%) | 20 (8.2%) |  |
| Tumor_size: |  |  |  | 0.028 |
| ≤2cm | 102 (29.2%) | 20 (19.0%) | 82 (33.6%) |  |
| ≤5cm | 161 (46.1%) | 52 (49.5%) | 109 (44.7%) |  |
| >5cm | 51 (14.6%) | 18 (17.1%) | 33 (13.5%) |  |
| Unknown | 35 (10.0%) | 15 (14.3%) | 20 (8.2%) |  |
| RNE: |  |  |  | 0.439 |
| ≥16 | 132 (37.8%) | 36 (34.3%) | 96 (39.3%) |  |
| 1-15 | 217 (62.2%) | 69 (65.7%) | 148 (60.7%) |  |
| Stage IB: |  |  |  | 0.048 |
| T1N1M0 | 132 (37.8%) | 31 (29.5%) | 101 (41.4%) |  |
| T2N0M0 | 217 (62.2%) | 74 (70.5%) | 143 (58.6%) |  |
| Benefit: |  |  |  | 0.79 |
| Benefit | 194 (55.6%) | 60 (57.1%) | 134 (54.9%) |  |
| Non-benefit | 155 (44.4%) | 45 (42.9%) | 110 (45.1%) |  |


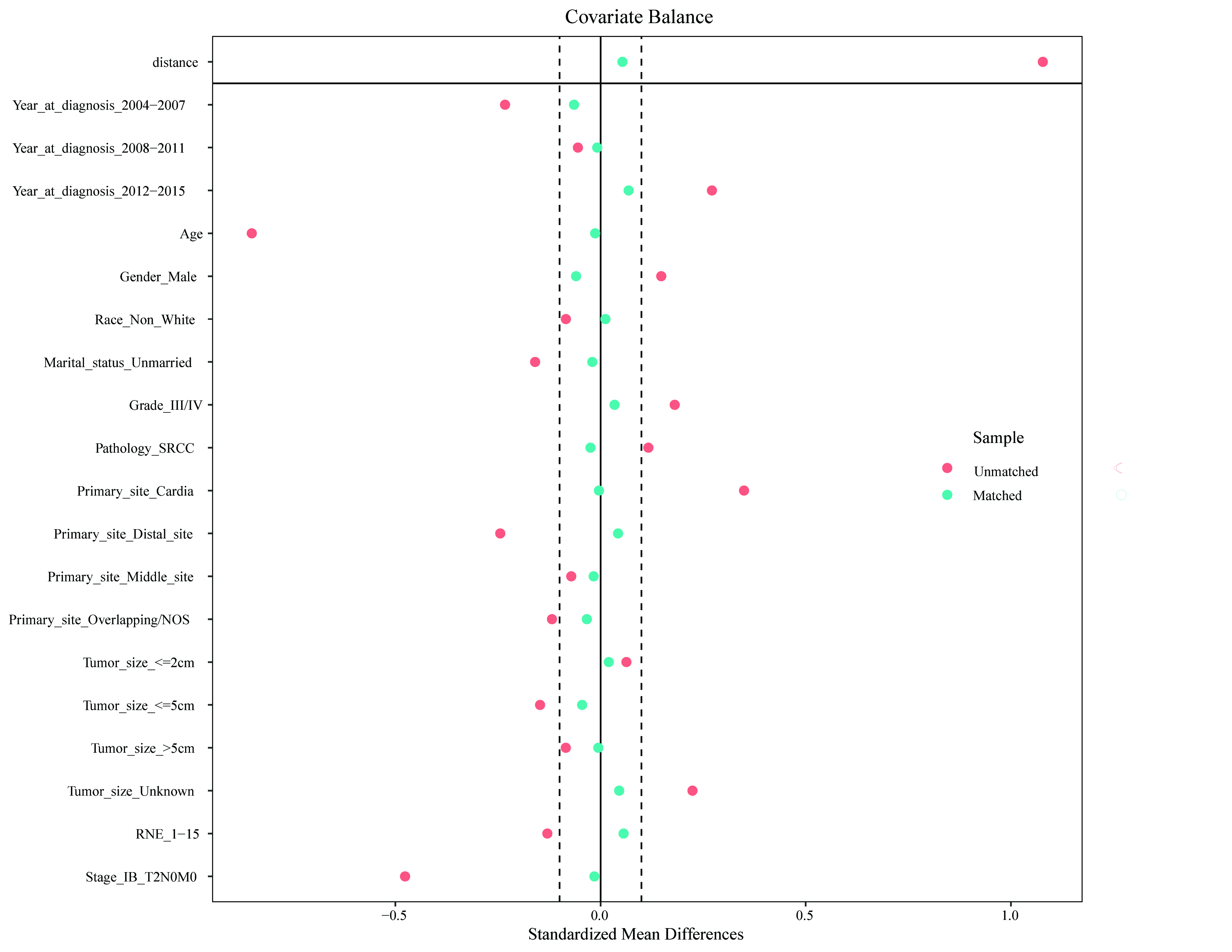


**Figure S1: The mean difference between the two cohorts.**


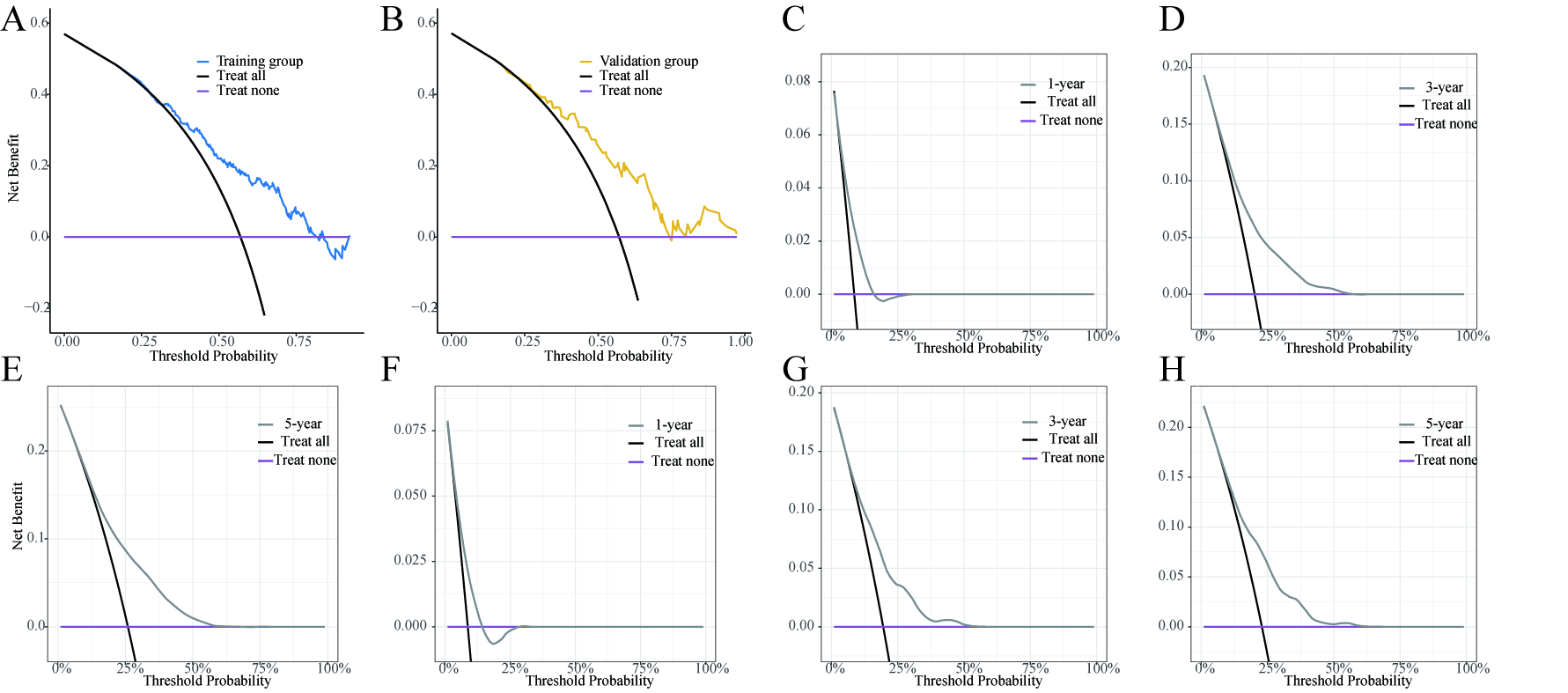


**Figure S2: DCA curves of the benefit nomogram in the training (A) and validation (B) cohort. DCA curves of the prognostic nomogram in the training (C-E) and validation (F-H).**
